# The FHJ debate: The NHS is failing to provide services for patients with symptom-based disorders

**DOI:** 10.1016/j.fhj.2025.100482

**Published:** 2025-12-15

**Authors:** Chris Burton, Benjamin Ellis

**Affiliations:** a Professor of Primary Medical Care, School of Medicine and Population Health, University of Sheffield, 30 Regent Street, Sheffield, South Yorkshire, UK; b Consultant Rheumatologist, Clinical Director for Outpatient Transformation, Imperial College Healthcare NHS Trust

## Proposition: The NHS is failing to provide services for patients with symptom-based disorders

*Chris Burton*

‘Symptom-based disorders’ is a relatively new term to encompass a range of syndromes and persistent physical symptoms in which diagnosis is based on symptoms rather than biomedical tests. These disorders include fibromyalgia and irritable bowel syndrome (one of many ‘disorders of the gut–brain axis’),^1^ and functional neurological disorders have been recognised for many years. They are common and are associated with significant impact at both individual and societal levels. Importantly, they are becoming increasingly understood as disorders of brain–body signalling,^2^ including nerve, immune and behavioural regulatory processes.^3^

I will argue that the NHS is failing patients with symptom-based disorders in multiple ways. However, in describing these ways, I hope to suggest opportunities for change. I suggest four ways that the NHS currently fails these patients: (1) failing to provide authoritative assessment / diagnosis / formulation, (2) failing to address the structural stigma which patients face, (3) failing to ‘join the dots’ across or between services, and (4) failing to provide therapeutic optimism.

## Failing to provide authoritative assessment and diagnosis

While symptom-based disorders may not (currently) have definitive pathology or diagnostic tests, many do have diagnostic criteria that have been developed through research and can be applied in practice.^3^ However, these disorders also sometimes occur with sufficiently ambiguous symptoms that a proper differential diagnosis is a necessary part of safe assessment and diagnosis.^4^ The increasing specialism of medicine, along with fear of incorrect diagnosis, means that for many practitioners the safest route to diagnosis is the opinion of someone more specialised. If there is the capacity to provide that, it is fine. But in the NHS, that capacity often isn’t there. That means at each level (advanced clinical practitioner, GP, physician, expert in symptom-based disorders), there needs to be clarity about how safe diagnosis can be promoted and supported. Trickle-down diffusion of knowledge can help; explicit knowledge mobilisation may help more. While the role of the physician specialist will not be to diagnose every case (especially with the more common disorders), our patients would get a better deal if services were better at mobilising and disseminating skills (and confidence) so that more people could make those assessments and diagnoses in a safe and constructive way. Authoritative assessment and diagnosis are key elements of recognition and validation of patients’ conditions. And without them, we reduce patients’ opportunity to recover.^5^

## Failing to address the structural stigma faced by patients

People with symptom-based disorders commonly report negative experiences of healthcare. This includes stigma: ‘a process or experience characterised by exclusion, rejection, blame, or devaluation that results from … an adverse social judgement about a person identified with a particular health problem’.^6^ Stigma occurs towards individuals with symptom-based disorders and can be directed towards the condition, the person or their behaviour, and takes many forms within healthcare consultations.^7^ To reduce this form of iatrogenic harm, services need to consider how we often unwittingly perpetuate stigma and address the negative ways in which symptom-based disorders and patients with them are viewed. While more could be done in medical education,^8^ we need to change the way we think at all levels.

## Failing to ‘join the dots’

Medicine loves a single answer that explains everything. From Dr Conan Doyle to Dr House, the appeal of an elegant and simple solution has always been strong. But with symptom-based disorders, we need to be willing to embrace multiple elements and to think beyond clinical silos and disease-specific pathways. People with symptom-based disorders commonly have symptoms in multiple body systems: it is only by thinking neyond specialty boundaries that these become apparent. Current conceptualisations of symptom-based disorders are genuinely biopsychosocial (with all three components being important, rather than just using the term as a euphemism for ‘not really medical’).^9^ The NHS has a range of coaching and therapeutic services that can be useful for many patients with symptom-based disorders, but we need to get better at actively referring people in a positive way: guiding people towards other services, rather than signposting away from mine.

## Failing to provide therapeutic optimism

Finally, we don’t do enough to promote therapeutic optimism. Many people with symptom-based disorders are stuck, without an authoritative diagnosis or formulation, stigmatised and bounced between services. However improvement, and sometimes recovery, are possible. This can be difficult for patients to hear in the current climate of illness identity politics and social media bubbles, but it is important. Hope – the possibility that things can be different – is important. Our recent trial of extended medical consultations for people with multiple physical symptoms found statistically significant differences in symptoms sustained for at least 9 months after the end of treatment.^10^ The number needed to treat for twice the clinically important difference in symptoms was five. We can make a difference.

## Opposition: The NHS is not failing to provide services for patients with symptom-based disorders

*Benjamin Ellis*

To claim that the NHS is failing patients with symptom-based disorders fundamentally misunderstands what people with these conditions require, and what our health service actually provides. To support this motion and declare failure is a dangerous oversimplification that undermines the very structures best positioned to help millions of people who need it most.

Symptom-based disorders include conditions like fibromyalgia, chronic fatigue and irritable bowel syndrome, which present with persistent, distressing symptoms that tend to cluster and are not well explained or treated by the dominant medical model. These account for a high proportion of primary care consultations and up to a third of referrals to specialist care.

In focusing on ‘services’, this motion presents a fundamental misunderstanding of what constitutes appropriate care here. To suggest that we need dedicated ‘fibromyalgia services’ or ‘irritable bowel syndrome clinics’ ignores the reality that those affected often experience many different bodily, cognitive and psychological symptoms. Creating organ-specific services would fragment care precisely when an integrative approach is needed most.

The evidence is clear. People with symptom-based disorders don’t benefit from being ‘sliced and diced’ by traditional medical models focused on identifying and treating specific pathological processes. What people need – what the NHS is well placed to deliver and increasingly provides – are services emphasising long-term, trusting relationships with healthcare teams, clear explanations of these confusing, distressing and disabling symptoms, and support to achieve personal goals and priorities, in line with people’s values and beliefs.

Critics conveniently ignore the transformation already underway within NHS in England, set out in the 2019 Personalised Care strategy, as part of the NHS Long Term Plan. This introduced new roles, including social prescribing link workers and health and wellbeing coaches, which are revolutionising support for people affected with symptom-based disorders. Declaring failure of this model so early on risks missing the opportunity to put these successes at the heart of the new NHS Ten Year Plan, which emphasises ‘from hospital to community’ and ‘from treatment to prevention’ as two of its big three shifts.

People with symptom-based disorders, such as fibromyalgia, are already benefiting from this new approach. In north-west London, we showed the value of including health and wellbeing coaches in a primary care-based model of care.^11^ A project run by the University of Southampton and Keele University has developed an intervention to reduce pain-related distress, with a key role for social prescribers.^12^ Around Bradford, the Rethinking Pain project has employed health coaches, social prescribers and community partners to transform care for people with chronic pain.^13^ My opponent in this debate has himself demonstrated that a straightforward, symptom-focused intervention delivered within NHS primary care ‘led to sustained improvement in multiple and persistent physical symptoms’.^10^

These aren’t niche experiments – they demonstrate that the machinery to support people with symptom-based disorders is already in place. These successes aren’t possible in spite of NHS structures, but because of them – our integrated primary care system provided the ideal platform for designing, delivering, evaluating and spreading these practices.

To declare the NHS ‘failing to provide services’ is to wilfully ignore this broad-scale transformation in how we now understand and support people with these conditions.

NHS primary care, despite facing unprecedented pressures, remains remarkably resilient. General practice continues to provide the continuity, accessibility and holistic approach that patients with symptom-based disorders need. Integration of mental health services, social care and third-sector organisations – coordinated through primary care – creates exactly the comprehensive support network these patients require. The sheer prevalence of these conditions shows that the solution must be in general practice, not in creating more specialist services that would inevitably become overwhelmed.

Yes, provision remains inconsistent, quality varies and more clinicians need to develop the skills to embed these approaches. These are arguments for acceleration, not evidence of failure. The infrastructure exists, the evidence base is growing, and innovative practices are expanding across the country.

The reality is that the NHS, through its evolved primary care model and innovative services, provides exactly the kind of integrated, person-centred care that robust evidence shows people need. The NHS is pioneering new approaches, implementing evidence-based interventions, and transforming care delivery. Rather than declaring failure, we need to celebrate, support and expand what’s already working. The solution is to build on foundations that, despite challenges, remain fundamentally sound.

What do you think? Vote at https://forms.office.com/e/0C2DCuf9A7 until 1 February 2026. Do you like the debate feature? Send us your thoughts to fhj@rcp.ac.uk.

## CRediT authorship contribution statement

**Chris Burton:** Writing – review & editing, Writing – original draft, Conceptualization. **Benjamin Ellis:** Writing – review & editing, Writing – original draft, Conceptualization.

## Declaration of competing interest

The authors declare the following financial interests/personal relationshipswhich may be considered as potential competing interests. Christopher Burton reports that financial support was provided by the National Institute for Health and Care Research. Christopher Burton has patent with royalties paid to The ABC of Medically unexplained Symptoms. If there are other authors, they declare that they have no known competing financial interests or personal relationships that could have appeared to influence the work reported in this paper.
